# Reconstruction of an Integrated Genome-Scale Co-Expression Network Reveals Key Modules Involved in Lung Adenocarcinoma

**DOI:** 10.1371/journal.pone.0067552

**Published:** 2013-07-11

**Authors:** Gholamreza Bidkhori, Zahra Narimani, Saman Hosseini Ashtiani, Ali Moeini, Abbas Nowzari-Dalini, Ali Masoudi-Nejad

**Affiliations:** 1 Laboratory of Systems Biology and Bioinformatics (LBB), Institute of Biochemistry and Biophysics, University of Tehran, Tehran, Iran; 2 Department of Algorithms and Computation, College of Engineering, University of Tehran, Tehran, Iran; 3 School of Mathematics and Computer Science, University of Tehran, Tehran, Iran; University of Colorado School of Medicine, United States of America

## Abstract

Our goal of this study was to reconstruct a “genome-scale co-expression network” and find important modules in lung adenocarcinoma so that we could identify the genes involved in lung adenocarcinoma. We integrated gene mutation, GWAS, CGH, array-CGH and SNP array data in order to identify important genes and loci in genome-scale. Afterwards, on the basis of the identified genes a co-expression network was reconstructed from the co-expression data. The reconstructed network was named “genome-scale co-expression network”. As the next step, 23 key modules were disclosed through clustering. In this study a number of genes have been identified for the first time to be implicated in lung adenocarcinoma by analyzing the modules. The genes EGFR, PIK3CA, TAF15, XIAP, VAPB, Appl1, Rab5a, ARF4, CLPTM1L, SP4, ZNF124, LPP, FOXP1, SOX18, MSX2, NFE2L2, SMARCC1, TRA2B, CBX3, PRPF6, ATP6V1C1, MYBBP1A, MACF1, GRM2, TBXA2R, PRKAR2A, PTK2, PGF and MYO10 are among the genes that belong to modules 1 and 22. All these genes, being implicated in at least one of the phenomena, namely cell survival, proliferation and metastasis, have an over-expression pattern similar to that of EGFR. In few modules, the genes such as CCNA2 (Cyclin A2), CCNB2 (Cyclin B2), CDK1, CDK5, CDC27, CDCA5, CDCA8, ASPM, BUB1, KIF15, KIF2C, NEK2, NUSAP1, PRC1, SMC4, SYCE2, TFDP1, CDC42 and ARHGEF9 are present that play a crucial role in cell cycle progression. In addition to the mentioned genes, there are some other genes (i.e. DLGAP5, BIRC5, PSMD2, Src, TTK, SENP2, PSMD2, DOK2, FUS and etc.) in the modules.

## Introduction

Lung cancer is the major cause of cancer-related deaths in the world and is not easily diagnosed. At most, 15% of patients sustain life for no more than five years [1]. Lung cancer is categorized into two groups i.e. small-cell lung cancer (SCLC) that includes 20% of lung cancers and non small cell lung cancer (NSCLC) that comprises 80% of lung cancers. NSCLC is shown to initiate from lung epithelial cells that leads to various histological subvarieties including adenocarcinoma (ADC), large cell carcinoma (LCC) and squamous cell carcinoma (SCC). The incidence of ADC subtypes has relatively increased in the last few decades [2].

The variation in the human genome is diverse, including alterations in the human karyotype, point mutations, single nucleotide polymorphisms (SNPs) and so forth. The duplication, insertion and deletion variations within the range of one kilo-base to several mega-bases that wildly take place in human and other mammalian genomes are termed copy number variations (CNVs). Although there are powerful repair mechanisms in the human genome, the odds of CNVs are high in such a way that they are 100–10000 times higher than point mutations [3]. Recently, the role of CNV in various cancers has been thoroughly investigated in many ways [4].

Array based comparative genomic hybridization (array-CGH) is capable of supplying high-resolution identification of CNVs. Single nucleotide polymorphism arrays (SNP array) can quantify cancer-specific loss of heterozygosity (LOH) and CNV with high precision in a genome-wide manner [5].Through the use of the two approaches, several genomic regions frequently showing DNA gains (3q, 5p, 7q, 8q, 11q and 16p) and losses (3p, 4q, 5q, 6q, 8p 9p and 13q, 17q) have been detected in NSCLC patients. Genetic aberrations in NSCLC are potently associated with tumor histology and a comparison of the two histological subtypes of squamous cell carcinoma and adenocarcinoma has disclosed clear dissimilarities in the recurrence of genomic aberrations [6]. Being influenced by tumor subtype, gender, ethnicity and exposure to carcinogens (e.g. smoking, radon gas, asbestos and cooking oil fumes), built up genomic alterations apply different tumorigenic mechanisms bringing about triggering of oncogenic signaling pathways and unrestrained tumor growth and metastasis [7].

Previous studies on NSCLC, concentrating on oncogenic point mutations, have recognized repetitious mutations that lead to aberrant activation of EGFR, KRAS, PIK3CA, ERBB2, BRAF, and some other genes. In addition, inactivating point mutations and deletions in TP53, STK11, NF1, CDKN2A and PTEN have been demonstrated. Mutations in several tyrosine kinase genes including PDGFRA and KDR have also been identified. Compared with lung adenocarcinoma, the range of genetic alterations in lung squamous cell carcinoma is less known [8].

Recently, genome-wide association studies (GWAS) have proven that three human genomic regions in chromosomes 5p15, 15q25, and 6p21 are associated with vulnerability to lung cancer in European and American populations. Besides, 3q29 and 18p11.22 are associated with susceptibility to lung cancer in Korean population [9].

The modern advancement of cDNA and oligonucleotide microarray analysis has enabled us to broadly analyze gene expression profiles in NSCLC cells and classify lung cancers at the molecular scale [10]. Gene expression profiling has given us many genes over- or down-expression in different disease states, but again a small number of the genes have been shown to be clearly functionally relevant to the tumorigenic procedure [11].

Our goal was to construct a “genome-scale co-expression network” for lung adenocarcinoma using all available and relevant data. The rationale behind our idea is the fact that simultaneous use of SNP array, gene expression microarray, array-CGH, CGH, GWAS and gene mutation data can give a more comprehensive understanding of the whole genome of the cancerous cells. Inasmuch as the genomic variations are different in various NSCLC subtypes, we focused on lung adenocarcinoma to achieve more precise results. In this study, through the integration of data obtained from the analyses mentioned above, it is feasible to deduce an integrated genome wide perspective of the mutated genes, the gene dosage aberrations and their effect on gene expression. Such achievements may help us in identifying the significant genes in lung adenocarcinoma. Another main aim of our study was to find the key modules in lung adenocarcinoma. Accordingly, through clustering of a “genome-scale co-expression network”, lung adenocarcinoma modules were revealed. Another objective achieved by analyzing the modules was to identify the significant genes implicated in lung adenocarcinoma.

## Materials and Methods

**Figure 1:**
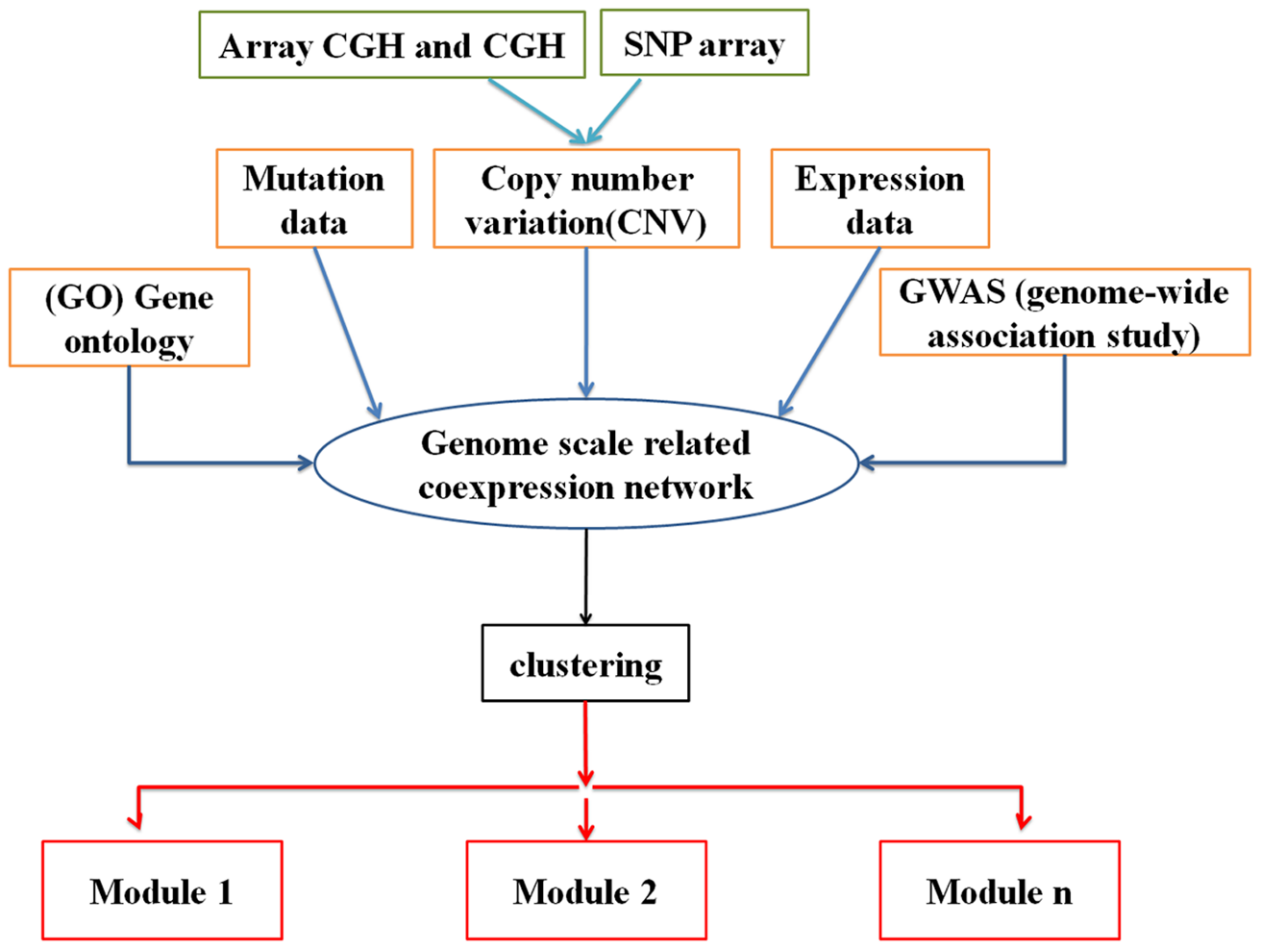
A framework with the purpose of “genome-scale co-expression network” construction and corresponding analysis.

Figure 1 depicts a framework for construction of a “genome-scale co-expression network” in lung adenocarcinoma which includes different integrated data. Gene mutations, GWAS, array-CGH, CGH and SNP array data were used to identify the loci and the genes that exist in lung adenocarcinoma, with high precision. In other words, through integration of the mentioned data, lung adenocarcinoma was examined in genome-scale. Subsequently, gene expression microarray data were used to integrate with other data in order to construct “genome-scale co-expression network”. In the next step, using clustering, the key modules in lung adenocarcinoma were revealed and analyzed.

### Identification of the genes located on CNV segments in lung adenocarcinoma

Datasets for SNP microarray (SNP array) related to adenocarcinoma were obtained from the NCBI Gene Expression Omnibus (GEO) (http://www.ncbi.nlm.nih.gov/geo). The accession numbers were GSE33848 [12] and GSE36363 [13], and there were 216 samples in total. The applied datasets have Affymetrix Genome-Wide Human SNP 6.0 array platform. The genome-wide Human SNP Array 6.0 consists of SNPs and CN probe sets related to two enzyme sets, namely Nsp and Sty.

The information regarding CNV regions was obtained by Affymetrix^®^ Genotyping Console™ software (GTC). A number of SNPs and CN probe sets are merely located on fragments made by one of the enzymes, whereas the rest of SNPs and CN probe sets are located on fragments made by both of the enzymes. Genotyping Console 4.0 (GTC 4.0) harbors in its interface the possibility of choosing between genotyping SNP 6.0 array data with the Birdseed (v1) and the Birdseed (v2) algorithms. Birdseed v2 applies EM to generate a maximum likelihood fit of a 2-dimensional Gaussian mixture model in A*B space. V1 applies SNP-specific models merely as an initial condition that allows the Expectation-Maximization (EM) fit to wander more freely leading to probable misleading of the clusters. On the other hand, Birdseed v2 uses SNP-specific priors in likelihood as Bayesian priors in addition to initial priors. This is considered as an advantage over v1 because the EM cannot freely wander with such constrains. SNP 6.0 CN/LOH analysis takes the advantage of the BRLMM-P+ algorithm, which is comparable with BRLMM-P, though with a few dissimilar parameters. GTC 4.0 was run with its default parameters. After GTC 4.0 run, only the regions (loci) that had been observed in at least 15% of the cancerous samples, were selected. Eventually, we determined the genes located in the mentioned CNV regions on the basis of NCBI Gene (http://www.ncbi.nlm.nih.gov/gene).

Array-CGH and CGH datasets related to adenocarcinoma were obtained from the source (http://www.cghtmd.jp/CGHDatabase). The selected cell lines are RERF-LC-MS, ABC-1, RERF-LC-OK, PC-14, HUT-29, SK-LC-3, VMRC-LCD, 11-18 and A549, which are related to lung adenocarcinoma.

### Gene mutation and GWAS data

Gene mutation data regarding adenocarcinoma were obtained from HGMD (http://www.hgmd.cf.ac.uk/ac/indedx.php), RCGDB (http://rcgdb.bioinf.uni-sb.de/MutomeWeb/) and COSMIC (Catalogue of Somatic Mutations in Cancer) (http://www.sanger.ac.uk/genetics/CGP/cosmic/add_info/). GWAS data were gathered from a literature search, from which, some genes and loci were chosen [14], [15], [16], [17].

### Gene expression microarray data

Datasets for Gene expression microarray of adenocarcinoma were obtained from GEO database. For including the most number of genes possible and in order to maximize the number of databases, the Affymetrix Human Genome U133 plus 2.0 Array platform datasets were used to build the co-expression network and various datasets were applied to denote multiple perturbed states in adenocarcinoma. In total, 158 samples were chosen from three datasets i.e. GSE12667 [18], GSE10245 [19] and GSE28571 [6]. GSE28571 and GSE10245 datasets also involve histological subtypes such as large cell carcinoma and squamous cell carcinoma in addition to adenocarcinoma, nonetheless, only adenocarcinoma samples were chosen.

### Reconstruction of “genome-scale co-expression network”

There are many existing methods to reconstruct a biological network from microarray data. The Methods based on machine learning, for example Bayesian network [20], [21] and clustering algorithms, or methods based on information theory [22], [23], [24] are some of methods used in reconstruction of gene regulatory networks. ARACNE [24] is one of the popular statistical algorithms for the reconstruction of accurate cellular networks using microarray expression profiles. ARACNE is also flexible to work on complex mammalian cell data, and it uses statistical methods to eliminate indirect links between genes. It is, therefore, fast and efficient enough to reconstruct “genome-wide co-expression networks”.

Candidate gene-gene interactions are estimated by pairwise analysis of the expression profile using the mutual information. I (g_i_, g_j_) = I_ij_ is an information theoretic measure of relatedness which is zero if P(g_i_) and P(g_j_) are independent variables, i.e. P(g_i_, g_j_) = P(g_i_).P(g_j_). Choosing an appropriate threshold for the mutual information can determine which gene expressions can be considered related to each other. The mutual information in ARACNE is computed using formula 1, where x_i_ and y_i_ represent expression levels and P(x_i_) and P(y_i_) represent the probability that X = x_i_. The mutual information threshold can be imported as an input of ARACNE using a P-value parameter. This factor alone suffers from the problem of considering indirect interactions.
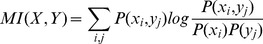
(1)


Then. ARACNE removes candidate indirect interactions using Data Processing Inequality (DPI) property, which is a well-known information theoretic measure. If formula 2 holds for the mutual information values between g_1_, g_2_, and g_3_, then DPI states that genes g_1_ and g_3_ only have relations because of their relations with the third gene, g_2_, and they do not have a direct relation between themselves.
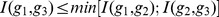
(2)


ARACNE was used for construction of a co-expression network for the expressed data. For construction of a “genome-scale co-expression network”, the co-expression network was constructed according to the genes obtained from gene mutation data, GWAS data, array-CGH, CGH and SNP-array analysis. These genes (obtained through data integration) were used as a hub at the entrance of ARACNE so that the co-expression network is constructed on the basis of these genes.

### Clustering of “genome-scale co-expression network”

Topological characteristics of the co-expression network were examined by Cytoscape 2.8.3 [25] and for clustering, ClusterONE [26] and MCODE [27] were used. ClusterONE, a Cytoscape plugin for clustering, was used as the clustering method in this section. This algorithm is fast and can be run in a command-line mode, which does not need to load the large genome wide network in Cytoscape. ClusterONE is designed to find densely connected subgraphs of a network by maximizing edges (weights) within a cluster and minimizing edges (weights) between different clusters. It allows the overlapping of subgraphs (clusters), which are necessary in gene co-expression networks, since a gene may take part in more than one functional module.

MCODE is another method for clustering that was used herein. MCODE is a clustering algorithm, which can be used for directed or undirected graphs. With our undirected co-expression graph, we can summarize MCODE algorithm in three steps: vertex-weighting, complex prediction, and the optional post-processing phase. The vertex-weighting function is defined as the product of the vertex core-clustering coefficient and the highest k-core level of the immediate neighborhood of the vertex. This weighting scheme defines a measure of local density for a vertex's neighborhood. In the second stage, complexes with high vertex weight are used as seed and the complex neighbor vertices are checked to see if they are a part of this complex or not. This check is done using a weight threshold on the percentage weight vertex, which, is away from the weight of the seed vertex. This process is repeated until no other vertex can be added to this complex. In this way, complexes are detected. In the third phase, a post-processing is done in which some complexes may be removed (if they do not have a minimum degree of 2), and some complexes may enlarge according to a given fluff parameter. MCODE main algorithm – until step 2 – results in non-overlapping subclusters, which are not suitable for clustering co-expression networks in which a gene may take part in more than one module. The only possible overlap in MCODE can happen in the third phase of the extending complexes.

## Results

### Mutated genes and identified genes by GWAS in lung adenocarcinoma

All 141 mutated genes selected from gene mutation databases were related to lung adenocarcinoma. In addition, 41 genes were chosen from the studies related to GWAS, which were previously published. The number of the genes reached 181 unique genes in total, as listed in the Table S1.

### CNV analysis

**Figure 2:**
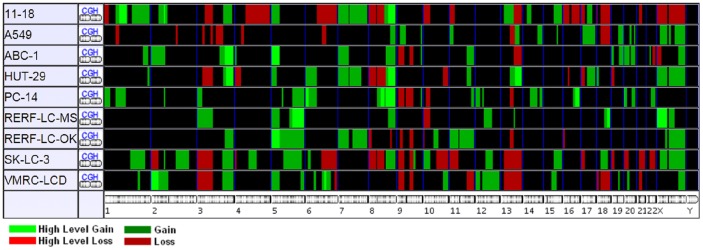
Gain and loss comparison obtained from CGH and CGH-array analysis in different lung adenocarcinoma cell lines.

Our studies, along with several other ones, have shown that some of the regions including loss and gain in all types of NSCLC are quite different. For this reason, we focused on lung adenocarcinoma to get better results, considering a subtype from NSCLC. CNV analysis was done through the integration of SNP-array, CGH and array-CGH data. Only those loci from gain and loss were chosen which were already present in both. Array-CGH data were obtained from CGMD database with nine lung adenocarcinoma cell lines. Figure 2 depicts the regions having gain (duplication) and loss (deletion) in different cell lines. Gain and loss items were chosen from only those common in at least 40 percent of all cell lines. Gain items were 2p13, 3q25-29, 5p12-15, 5q23, 5q33-35, 7p11-22, 8q22-24, xq12-28, xp11-22 and 4q13, and loss items were 3p12-21, 8p22-23, 9p13, 9p21-23, 9q13, 9q21 and 13q21-34.

**Figure 3:**
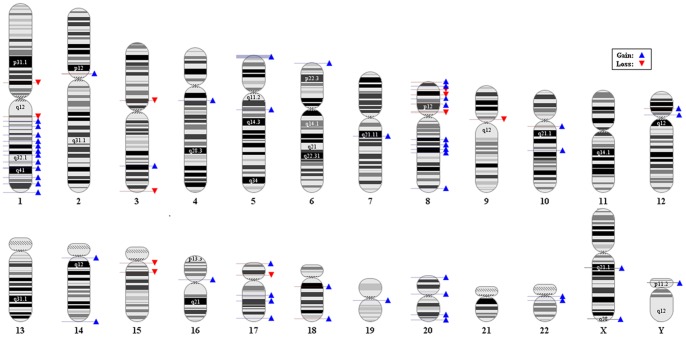
A schema of the loss and gain obtained from SNP-array analysis.

SNP-array analyses were performed using Genotyping console 4.0 and Gain and loss items were chosen in such a way that only those common in at least 15 percent of all cell lines were collected. Gain items involved some regions of 1q, 2p11.2, 3q26.1, 5p15.33, some regions of 5q, 6p25.3, 7q21.11, 14q13.2, some regions of 8, 10q11-22, 12p11-12, some regions of 14q, 15q11.2, 16p11, some regions of 17p and 18q, 19q12, some regions of 20, 22q11, some regions of Xq and Yp11.2 and loss items included 8p11.22, 9p11.2, 3q29, 17p11.2, 1q21.1-2, 3p12.3, some regions of 15q and xp22. Figure 3 shows gain and loss regions caused by SNP-array analysis. Then, another filtering was carried out to increase the precision of the loci including gain and loss items, so that finally those loci were chosen that existed among the output of array-CGH, CGH and SNP-array analyses. These loci, along with their genes downloaded from Entrez Gene, are provided in Table S2. The total number of all these genes is 3652.

### Reconstruction of “genome-scale co-expression network”

For construction of a “genome-scale co-expression network”, using ARACNE, the co-expression network was constructed according to the genes obtained from gene mutation data, GWAS data, array-CGH and SNP-array analysis in such a way that the genes obtained from gene mutation data and GWAS data (Table S1) were merged with the genes obtained from CNV analysis (Table S2). After the merger of the obtained 3833 genes, no more than 1588 ones included Probe and GO term and we only used those genes, which contained probe and GO term. These 1588 genes (Table S3) were used as hubs at the entrance of ARACNE so that the co-expression network is constructed on the basis of these genes. This co-expression network was named “genome-scale co-expression network”, because the co-expression network was constructed on the basis of the genome-scale data. We used ARACNE with the following parameter settings in Table 1.

**Table 1:** Parameter settings used for ARACNE.

Parameter	Value
List of probes for which a subnetwork will be constructed (-s)	1588 probes (Table S13))
P-value for MI threshold (-p)	9.14511467E-7 (0.05/number-of-probes)
Algorithm (accurate|fast : -a)	Accurate
Kernel width (-k)	default: determined by program
MI threshold (-t)	0

Since we used “–s” option (Table 1) , the resulting components each has at least one gene from our manually created gene set, and the components not including these genes were removed from the results. This is because we didn’t want to interpret any result that was not included in our gene set, therefore, this helped to reduce the computation needed to get our final co-expression network. ARACNE was implemented with P-values of 0.03 and 0.05, however, it led to no significant difference in the final clustering results and this is why from this point ahead, the results with P-value of 0.05 were employed.

The resulting ARACNE co-expression network on our microarray expression data of 54675 probes has 43058 nodes and 2015975 edges. This large network is comprised of 12 connected components as follows: One component (the largest) with 43031 nodes and 2015959 edges, eight components containing only 2 nodes (therefore 1 edge), two components with 4 nodes and 3 edges, and one component with 3 nodes and two edges.

### Clustering of “genome-scale co-expression network”

To analyze the results, we applied clustering on the “genome-scale co-expression network” generated by ARACNE. Clustering algorithms are used to find important sub networks or modules. The results were first obtained from clustering with MCODE but the results were not well-clustered because the clustering was performed on the basis of network topology regardless of the edge weight. Therefore, clusterONE was used which performs clustering on the basis of the edge weight (MI).

We ran ClusterONE with its default parameter settings on the basis of MI. ClusterONE output was 91 clusters or modules including 2237 genes (considering overlapping cluster genes). Some of the clusters turned out to be subclusters of other larger clusters, therefore omitting such subclusters the final number of clusters was reduced from 91 to 23, including 972 genes. Many of the genes are repeated in more than one cluster and overall, there were 450 unique genes out of 23 modules. These clusters are available in Table S4. The co-expression network is made around the axis of the hubs, where all the resulting modules contain at least on hub. For each module, the full data (probe ID, gene full name, synonym, GO…) are provided in Table S5.

## Discussion

**Figure 4:**
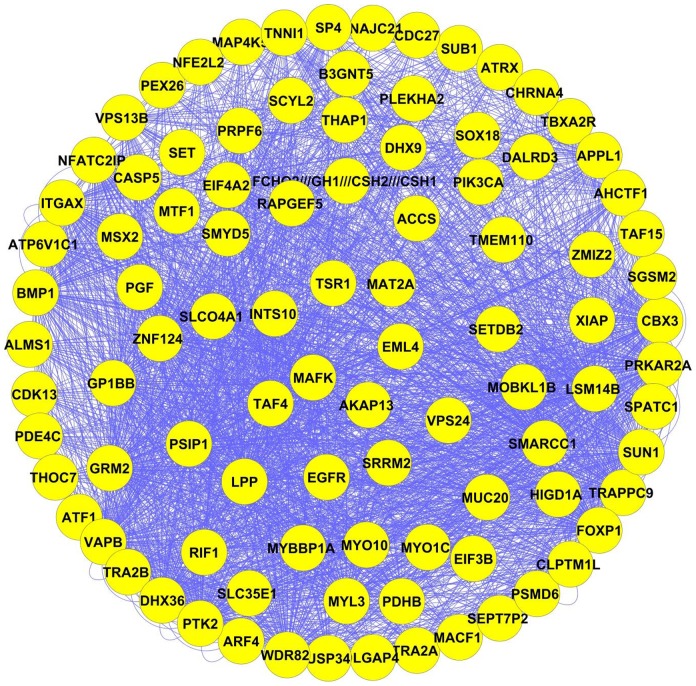
95 genes in Merged-module; each node denotes a gene in Merged module and edges depict co-expressed genes relationships.

**Figure 5:**
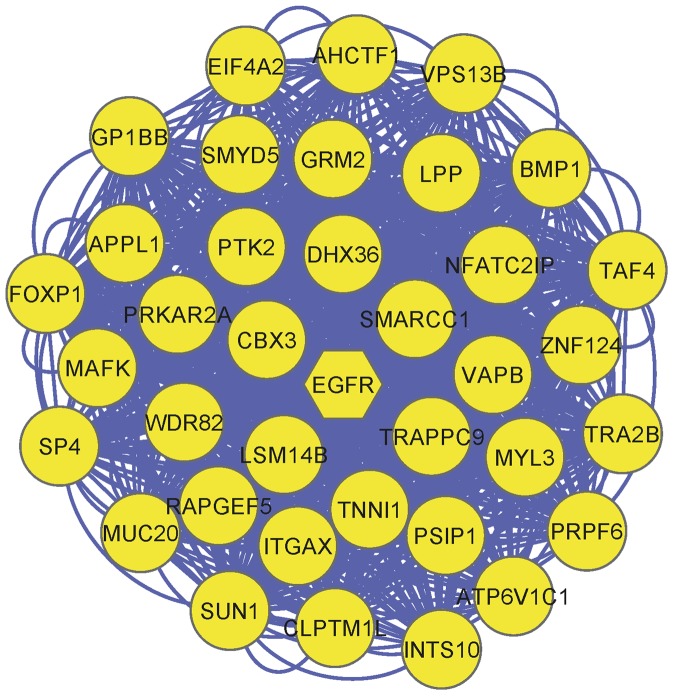
The first neighborhood of EGFR node in Merged-module.

Our results have detected 23 key modules in lung adenocarcinoma, which are accessible in detail in Table S5. Since EGFR signaling plays a key role in NSCLC, we have mainly chosen modules 1 and 22 (which contain EGFR) as representative modules for our discussion. The merger of the two modules with the most identical genes (named “Merged-module” in this study) is depicted in Figure 4 and Figure 5. Figure 5, illustrates the first neighborhood of an EGFR node in Merged-module whose genes were underlined. It is explained below that many of the identified genes play critical roles in different types of cancer. Additionally, other selected modules will be discussed briefly.

Various studies have shown that EGFR signaling plays a very important role in NSCLCs [28], [29], [30], [31], [32]. Since EGFR signaling is crucial for the cell survival and proliferation, it might be the main reason for tumor progression in NSCLC. EGFR signaling activates Ras/ERK, PI3K/Akt and STAT activation pathways. These three pathways are the main routes for the cell proliferation and survival [33], [34], [35]. Therefore, mutations that lead to excessive activation of these pathways may cause cancer. There are many reports on EGFR over-expression in NSCLC [30]. Hirish et al. [36], Mukohara et al. [31] and Rush et al. showed that the over-expression occurred in 62%, 78% and 98% of NSCLCs, respectively. Hirsh et al. via FISH [36], Gandi et al. via qPCR, CGH and FISH [29] showed a significant correlation between EGFR gene copy number increase with increasing EGFR expression. Knowing that the clustering was performed on the basis of MI, it is concluded that other genes present in the Merged-module are in accordance with the same expression pattern as that of EGFR. As such, all of these genes show over-expression in lung adenocarcinoma.

The PI3K-Akt pathway is deemed a potential regulator of the cell survival and proliferation. Some of the genes such as PIK3CA, TAF15, VAPB, Appl1, Rab5a, ARF4, and XIAP in Merged-module activate the PI3K-Akt pathway and are overexpressed in a manner similar to that of EGFR in lung adenocarcinoma. The Phosphoinositide-3-kinase catalytic alpha (PIK3CA) incearsed activity has been observed in a number of human cancer types such as breast, colon, liver, brain, stomach and lung [37]. TAF15 gene is also upregulated in liposarcoma [38]. XIAP protein over-expression has been identified in six NSCLC cell lines [39] and its inhibition triggers apoptosis in human lung adenocarcinoma A549 cells [40]. A genome-wide microarray analysis demonstrated that VAPB has been frequently overexpressed or amplified in breast cancer. Over-expression of VAPB in MCF10A-HER2 cells increases Akt phosphorylation [41]. Appl1 is known as an adaptor that takes part in cell signaling through interaction with different signaling molecules involving Akt, PI3-kinase (PI3K), Rab5, adiponectin receptor and TrkA [42], [43]. Rab5a being activated by Appl1 is significantly overepressed in ovarian cancer and is connected with lung, hepatocellular and stomach carcinomas [44]. Another gene in Merged module is ADP-ribosylation factor 4 (ARF4) that activates EGFR signaling has an anti-apoptotic function in human glioblastoma-derived U373MG cells [45]. Shigematsu et al. [46] and Sordella et al. [47] disclosed the activity of PI3K/Akt pathway in NSCLC. Herein, we have unraveled the over-expression of the above genes, which cause Akt over-activation. PIK3CA, XIAP and Rab5a have previously been reported in lung cancer and we have reported the effect of the rest of the mentioned genes for the first time.

Furthermore, two genes in Merged-module i.e. CLPTM1L and NFE2L2 are implicated in activation of the anti-apoptotic BCL2 family. The gain of CLPTM1L gene frequently occurs in the first stages of NSCLC. CLPTM1L is an overexpressed protein in lung tumor cells. Therefore, it denotes anti-apoptotic CLPTM1L function as a probable mechanism of vulnerability to lung tumorigenesis and resistance against chemotherapy [48]. NFE2L2 is a transcription factor up-regulated in the cell lines of pancreatic cancer, ductal adenocarcinoma [49] and NSCLC [50], [51]. We have also revealed the over-expression of these two important genes in lung adenocarcinoma.

In Merged-module, there are some transcription factors as follows: SP4, ZNF124, LPP, FOXP1, SOX18, MSX2 and NFE2L2. The mentioned transcription factors show the same expression pattern as that of EGFR in lung adenocarcinoma. The SP family members such as SP4 are responsible for the regulation of the expression of a few genes such as EGFR. They are connected to cancer cell proliferation, differentiation and metastasis [52], [53]. ZNF124 (ZK7) mRNA expression was detected in several human tissues such as a number of leukemia cell lines [54]. With High-resolution array-CGH, Kang et al. [55] and Choi et al. [56] have demonstrated the lipoma preferred partner (LPP) over-expression in NSCLC. FoxP1 (Proteins of the Forkhead-box family) has also been revealed to be expressed in many types of human malignant tumors, while it is along with metastasis. Elevated expression of FoxP1 has been demonstrated in NSCLC [57], hepatocellular carcinoma and breast cancer [58], [59]. SOX18, another gene from the above transcription factors, is essential for tumor-induced lymph-angiogenesis and metastasis and a probable target for anti-angiogenic therapy of human cancers [60]. MSX2 is likewise a critical regulator of embryonic development that is assumed to play a role in pancreatic and breast cancer [61]. Regarding the above explanation, these transcription factors, being related to proliferation, survival and angiogenesis, play an important role in lung adenocarcinoma. LPP, FOXP1 and NFE2L2 have previously been reported in lung cancer and we have reported the rest of the genes for the first time in lung adenocarcinoma.

There are some genes in Merged-module that their products take part in the transcription or splicing. These genes include SMARCC1, TRA2B, CBX3 and PRPF6 that show the same upregulation pattern as EGFR in lung adenocarcinoma and we have reported all the mentioned genes for the first time in lung adenocarcinoma. SMARCC1 protein upregulation has been reported in prostate cancer [62], colorectal cancer [63] and cervical intraepithelial neoplasia [62]. TRA2B over-expression was observed in endometrial cancers [64], Gastric cancer cells [65] and cervical cancer [66]. In addition, elevated levels of CBX3 expression in tumor stem cell (TSC)-enriched osteosarcoma cultures was detected [67]. PRPF6 is excessively expressed in the lymph node of lymphoma and is believed to be a probable target for tumor metastasis studies [68]. Regarding the roles of these factors in other cancers and their upregulation in Merged-module, it is possible to infer an important role for them in lung adenocarcinoma.

Four genes i.e. ATP6V1C1, MYBBP1A, MACF1 and MYO10 in Merged-module are connected to metastasis and migration. The increased levels of these gene expressions may be among the triggering factors of lung adenocarcinoma metastasis. The ATP6V1C1 has been shown to be involved in metastasis and multiple drug resistance. The ATP6V1C1 level is considerably high in oral squamous cell carcinoma [69], [70]. MYBBP1A is another gene in Merged module besides being a key regulator in tumor cell proliferation and migration e.g. head and neck squamous cell carcinoma [71]. MACF1 (ACF7) which is brought to the cell membrane via APC (adenomatous polyposis coli) in response to ERBB2 is the key factor for microtubule capture. MYO10 is the myosinΧ coding gene which is associated with Filopodia formation. This phenomenon has been detected in basal-type breast carcinoma [72]. We have discovered ATP6V1C1, MYBBP1A, MACF1 and MYO10 upregulation in lung adenocarcinoma and accordingly, it is concluded that these genes have an important role in lung adenocarcinoma metastasis and we have reported all the mentioned genes for the first time in lung adenocarcinoma.

Two other important genes i.e. GRM2 and TBXA2R in Merged-module being from the GPCR superfamily, showed over-expression in lung adenocarcinoma. PKA, another important gene found in GPCR signaling showed over-expression as well. Subtypes of GRM2 (mGluR2) are involved in the pathogenesis of diverse cancer types like breast cancer [73], medulloblastomas and gliomas in such a way that GRM2 is overexpressed in all of these cancers [74]. GRM2 keeps the activity of ERK and PI3K pathways. Both pathways are activated in response to EGF [75]. For this reason, we could observe EGFR-like GRM2 over-expression in lung adenocarcinoma. Breast tumor tissues express higher levels of TBXA2R [76]. One of the important factors in GPCR signaling is the regulatory subunit of PKA named PRKAR2A, which is overexpressed in lung adenocarcinoma [77], [78]. Elevated expression of Protein kinase A regulatory subunit has been found in primary tumors [79], AML and colorectal cancer [80]. We have reported all the mentioned genes for the first time in lung adenocarcinoma.

PTK2 [81], [82], PGF [83], [84], SLCO4A1 [85] and Cdc27 [86] are among the genes in Merged-module whose over-expression has been reported in different cancers including lung cancer and we have shown it as well. CDK13, BMP1, RNF13, MAT2A, CHRNA4 are also among the genes in Merged-module whose over-expression has been reported in different cancers, however, their over-expression has been demonstrated in lung adenocarcinoma in this study.

It is commonly accepted that the unleashed proliferation of cancerous cells is contingent on rising protein synthesis and the number of ribosomes. EIF3B that codes one of the EIF3A subunits is one of the Merged-module genes. EIF3B is a crucial part of the EIF3 complex that is implicated in tumor formation [87].Therefore, it is concluded that EIF3B plays a critical factor in enhancement of protein synthesis in lung adenocarcinoma. In some other modules, ribosomal protein genes named RPS20, RPL8 and RPS4X besides EIF3E, EIF3H, EIF1B, EIF4A2 and EIF2B5 are present. On the basis of these results, the current studies suggest that translation plays a pivotal role in tumor progression.

It is evident that cell cycle regulatory factors play vital roles in different types of cancer. We have observed similar expression patterns in modules 3 and 12 in the genes whose products play important roles in cell cycle regulation. These genes are CCNA2 (Cyclin A2), CCNB2 (Cyclin B2), CDK1, CDK5, CDC27, CDCA5, CDCA8, ASPM, BUB1, KIF15, KIF2C, NEK2, NUSAP1, PRC1, SMC4, SYCE2, TFDP1, CDC42 and ARHGEF9 (CDC42 regulator) that show over-expression in lung adenocarcinoma. These factors are all connected with cell cycle progression and their over-expression leads to tumor progression in lung adenocarcinoma.

Cyclin A2 (CCNA2) takes control of both S phase and G2/M transition regarding Cdk2 and Cdk1, respectively. In S phase, Cyclin A2 undertakes the initiation and progression regulation of DNA synthesis. Through the G2/M transition, Cyclin A2 has a crucial role in triggering Cyclin B1–Cdk1 activation [88], [89]. CDK1 andCDK2 are CDK partners of A- and B- cyclins. A-type cyclins are capable of binding both CDK1 and CDK2, however, B-type cyclins are associated with CDK1 [90]. CDCA5 (Sororin) joins the cohesin complex to regulate the segregation of sister chromatids. Sororin undergoes phosphorylation in mitosis. Sororin is one of the phosphoproteins and protein kinases such as Cdk1/cyclinB and ERK2 regulates its dynamic localization and function [91]. On the basis of what previously mentioned, CDCA5, CDK1, CCNA2 and CCNB2 functions are dependent to each other and we have identified a similar expression pattern in lung adenocarcinoma in a way that all were upregulated together. CDCA8 (Borealin), ASPM, BUB1, KIF15, KIF2C, NEK2, NUSAP1, PRC1, SMC4 and SYCE2 genes are involved in cell division. Since the functions of the mentioned genes are related, their expression patterns turned out to be similar in our study.

CDCA7 expression is controlled by E2F1 and MYC factors that play important roles in cell cycle[130]. CDCA7 is frequently overexpressed in human cancers such as chronic myelogenous leukemia and lung cancers [92]. We have unraveled its over-expression in lung adenocarcinoma. Cdc42 is overexpressed in many of primary lung cancer patients, and Cdc42 over-expression is significantly associated with high TNM stages and lymph node metastasis [93] and its role has recently been proved in lung cancer [94]. ARHGEF9 (Cdc42 guanine nucleotide exchange factor 9) selectively activates Cdc42 [95]. We have proved the over-expression of CDC42 and ARHGEER9 factors in lung adenocarcinoma.

## Conclusion

Various studies have shown that EGFR signaling plays a very important role in NSCLC [28], [29], [30], [31], [36]. Since, EGFR signaling is crucial for cell survival and proliferation; it might be the main reason for tumor progression in NSCLC. EGFR signaling activates Ras/ERK, PI3K/Akt and STAT activation pathways. These three pathways are the main routes for cell proliferation and survival [33], [34], [35], [96], [97], [98]. Four different reports have revealed an increase in PI3K/Akt and pSTAT3 activation pathways in NSCLC with EGFR mutation [46], [47], [99], [100]. Shigematsu et al. [46] and Sordella et al. [47] believe that EGFR mutation is specially exerting effects on PI3K/Akt and STAT3 having a minute effect on ERK activation (Ras/ERK pathway). But Mukohara et al. [31] through wet-lab and Bidkhori et al. [32] through *in silico* investigations have shown that in NSCLC, frequency levels of all three pSTAT3, pAkt and pERK are high. Besides, Amann et al. [101] and Vicent et al. [102] have shown that in NSCLC samples with EGFR mutation, pERK level is high. Our results prove that most of the overexpressed genes whose products take part in EGFR signaling pathway can activate PI3K/Akt pathway much more than the other two pathways. Furthermore, most of the mentioned genes products mainly belong to the PI3K/Akt pathway compared to the othe two pathways.

In this study, a number of genes have been identified for the first time to be implicated in lung adenocarcinoma. Some of these genes play pivotal roles in other cancer types such that they are considered as therapeutic targets. To manage and contain the investigation, we selected some modules among 23 modules as mentioned above. In addition, there are some genes in other modules that can have very important roles in lung adenocarcinoma, namely DLGAP5, BIRC5, PSMD2, Src, TTK, SENP2, PSMD2, DOK2, FUS among others. We suggest that the genes discussed here can also be used as potential leads for wet lab investigations.

## Supporting Information

Table S1 **A list from 181 genes obtained from gene mutation and GWAS data.**
(TXT)

Table S2 **Loci and genes obtained from CNV analysis.**
(TXT)

Table S3 **Final genes obtained from genome-scale analysis that used at the ARACNE input.**
(TXT)

Table S4 **23 key modules in lung adenocarcinoma.**
(CSV)

Table S5 **Full data for 23 key modules in lung adenocarcinoma.**
(ZIP)
